# Virome capture sequencing does not identify active viral infection in unicentric and idiopathic multicentric Castleman disease

**DOI:** 10.1371/journal.pone.0218660

**Published:** 2019-06-26

**Authors:** Christopher S. Nabel, Stephen Sameroff, Dustin Shilling, Daisy Alapat, Jason R. Ruth, Mitsuhiro Kawano, Yasuharu Sato, Katie Stone, Signe Spetalen, Federico Valdivieso, Michael D. Feldman, Amy Chadburn, Alexander Fosså, Frits van Rhee, W. Ian Lipkin, David C. Fajgenbaum

**Affiliations:** 1 Dana-Farber Cancer Institute, Massachusetts General Hospital Cancer Center, Boston, Massachusetts, United States of America; 2 Columbia University, New York, New York, United States of America; 3 Castleman Disease Collaborative Network, Philadelphia, Pennsylvania, United States of America; 4 Myeloma Center, University of Arkansas for Medical Sciences, Little Rock, Arkansas, United States of America; 5 Kanazawa University, Kanazawa, Japan; 6 Division of Pathophysiology, Okayama University Graduate School of Health Sciences, Okayama, Japan; 7 Oslo University Hospital, Oslo, Norway; 8 Hospital of the University of Pennsylvania, Philadelphia, Pennsylvania, United States of America; 9 Department of Pathology and Laboratory Medicine, Weill Cornell Medicine, New York, New York, United States of America; 10 Department of Medicine, University of Pennsylvania, Philadelphia, Pennsylvania, United States of America; University of North Carolina at Chapel Hill, UNITED STATES

## Abstract

Castleman disease (CD) describes a spectrum of heterogeneous disorders defined by characteristic lymph node histopathology. Enlarged lymph nodes demonstrating CD histopathology can occur in isolation (unicentric CD; UCD) sometimes accompanied by mild symptoms, or at multiple sites (multicentric CD, MCD) with systemic inflammation and cytokine-driven multi-organ dysfunction. The discovery that Kaposi sarcoma herpesvirus/human herpesvirus (HHV)-8 drives MCD in a subset of patients has led to the hypotheses that UCD and MCD patients with negative HHV-8 testing by conventional methods may represent false negatives, or that these cases are driven by another virus, known or unknown. To investigate these hypotheses, the virome capture sequencing for vertebrate viruses (VirCapSeq-VERT) platform was employed to detect RNA transcripts from known and novel viruses in fresh frozen lymph node tissue from CD patients (12 UCD, 11 HHV-8-negative MCD [idiopathic MCD; iMCD], and two HHV-8-positive MCD) and related diseases (three T cell lymphoma and three Hodgkin lymphoma). This assay detected HHV-8 in both HHV-8-positive cases; however, HHV-8 was not found in clinically HHV-8-negative iMCD or UCD cases. Additionally, no novel viruses were discovered, and no single known virus was detected with apparent association to HHV-8-negative CD cases. *Herpesviridae* family members, notably including Epstein-Barr virus (EBV), were detected in 7 out of 12 UCD and 5 of 11 iMCD cases with apparent correlations with markers of disease severity in iMCD. Analysis of a separate cohort of archival formalin-fixed, paraffin-embedded lymph node tissue by *In situ* hybridization revealed significantly fewer EBV-positive cells in UCD and iMCD compared to tissue from HHV-8-positive MCD and EBV-associated lymphoproliferative disorder. In an additional cohort, quantitative testing for EBV by PCR in peripheral blood during disease flare did not detect systemic EBV viremia, suggesting detection lymph node tissue is due to occult, local reactivation in UCD and iMCD. This study confirms that HHV-8 is not present in UCD and iMCD patients. Further, it fails to establish a clear association between any single virus, novel or known, and CD in HHV-8-negative cases. Given that distinct forms of CD exist with viral and non-viral etiological drivers, CD should be considered a group of distinct and separate diseases with heterogeneous causes worthy of further study.

## Introduction

Castleman disease (CD) describes a group of heterogeneous and poorly-understood lymphoproliferative disorders that share characteristic lymph node histopathology, including atrophic or hyperplastic germinal centers, prominent follicular dendritic cells, hypervascularization, polyclonal lymphoproliferation, and/or polytypic plasmacytosis. The disease was initially described in the 1950s by the pathologist Benjamin Castleman [1,2]. The disease was first classified as unicentric CD (UCD)—featuring a solitary enlarged lymph node with CD histopathology—or multicentric CD (MCD)—in patients with enlarged lymph nodes located in multiple body regions. Most UCD patients do not experience systemic symptoms and are successfully treated by removal of the enlarged node. In contrast, MCD involves constitutional symptoms, cytopenias, hepatosplenomegaly, fluid accumulation, and multiple organ system dysfunction [3–5]. In the mid-1990s, the HIV/AIDS epidemic led to the observation of MCD in immunocompromised patients [6]. Advances in molecular virology attributed these cases to co-infection with the gamma-herpesvirus Kaposi sarcoma herpesvirus /human herpesvirus (HHV)-8 [7,8], which evades host immunity in immunocompromised individuals and secretes a viral interleukin (IL)-6 analog in addition to directly stimulating host IL-6 production [9,10]. HHV-8-positive MCD patients often respond to rituximab and correction of the underlying causes of immunosuppression. Lastly, there is an additional cohort of MCD patients that do not test positive for HHV-8 infection using current clinical or histopathological assays and are referred to as idiopathic MCD (iMCD) because the etiology is unknown [11,12]. A subset of patients with iMCD may have a severe clinical presentation featuring thrombocytopenia, anasarca, myelofibrosis, renal dysfunction and organomegaly, now referred to as TAFRO syndrome[13,14].

Due to the overlapping clinical and pathologic similarities with HHV-8-positive MCD [11,15], it has been hypothesized that iMCD and UCD are in fact driven by viral infection—either HHV-8 that is not detected by current clinical diagnostic methods, a novel virus, or a known virus with a hitherto unnoticed association with CD. This hypothesis is supported by the episodic clinical course of iMCD with periods of remission and intermittent flares, which may correlate with a latent viral infection wherein reactivation may drive disease recurrence. HHV-8 belongs to the *Herpesviridae* family, which includes other lymphotrophic viruses such as Epstein-Barr virus (EBV), cytomegalovirus, and HHV-6, all of which are known to have similar cellular tropism to HHV-8 and are controlled through T cell immunity. EBV in particular, which joins HHV-8 in the gamma-herpesvirus subfamily, has been reported in association with iMCD and UCD in several case reports [16–18]. There are also case reports of associations with HHV-6 and hepatitis B virus (HBV) [17,19]; these smaller studies focusing on a limited repertoire of viral etiologies have not established a viral etiology for UCD or iMCD. However, neither a systematic study of known viruses, nor a search for novel viruses in iMCD or UCD has been reported. Furthermore, the search for viral associations with iMCD and UCD would have significant implications for diagnostics and treatment planning and remain an important frontier in understanding CD pathophysiology.

Recent advances in DNA and RNA sequencing technology have facilitated clinical studies of infectious diseases and viral discovery [20], presenting an opportunity to revisit the question of viral associations in CD. Prior to the advent of high-throughput methods, viral detection in the clinical setting was an iterative process involving a differential diagnosis based on known pathogens and a battery of tests with varying sensitivities. By contrast, modern high-throughput methods provide a less biased approach: a RNA amplification library is deeply sequenced and aligned to reference libraries for sequences that share identity with known pathogens [21,22]. The application of such a strategy does not require manual iteration of a differential diagnosis and instead enables an unbiased approach based on homology to known sequences from pathogen genome databases. Furthermore, utilization of the primary tissues in which infection is suspected can increase sensitivity in pathogen discovery, a principle demonstrated across many examples of clinical viral discovery in both human and animal studies [23].

Herein, we report results using modern techniques in viral discovery to study lymph node tissue from patients with CD. The virome capture sequencing for vertebrate viruses (VirCapSeq-VERT) approach, an RNA-hybrid capture, deep sequencing and bioinformatics pipeline designed for viral discovery [24], was applied to test the hypotheses that HHV-8, known viruses, or novel viruses are associated with CD.

## Materials and methods

All studies were approved by local ethics committees, where applicable, and by the University of Columbia’s or University of Arkansas for Medical Sciences’ institutional review board, and compliant with Health Insurance Portability and Accountability Act. All samples were de-identified prior to analysis, and analyzed anonymously.

### Patient materials

Fresh frozen lymph node samples from iMCD (n = 11), UCD (n = 12), HHV-8-positive MCD (n = 2), and malignant lymphoma without any known or suspected association or clinical suspicion of HHV-8 infection (n = 6) were collected by either core needle or excisional biopsy at diagnosis or later during flare as part of routine clinical treatment. From an entirely separate cohort of patients, archival formalin-fixed, paraffin-embedded lymph node tissue samples were collected from patients with iMCD (n = 12), UCD (n = 9), HHV-8-positive MCD (n = 4) and EBV associated post-transplant lymphoproliferative disorder (n = 4). Samples from patients with CD were assayed for HHV-8 infection at diagnosis or flare by either latency-associated nuclear antigen-1 (LANA-1) staining or PCR testing for HHV-8 DNA of biopsied lymph node tissue. All iMCD cases met the recently published international diagnostic criteria [13]. Flare is defined as presence of at least two minor criteria (including one abnormal laboratory test) in iMCD diagnostic criteria and/or CRP>10 mg/dL. All laboratory values, treatments, and sample collection dates were obtained from the patients’ medical records. Researchers were blinded to disease type for all sample analysis.

### VirCapSeq-VERT detection of viral sequences in frozen lymph node samples

For VirCapSeq-VERT analysis, 0.2 g was cut from each frozen tissue sample using a sterile scalpel. Solid optimum cutting temperature (OCT) compound was cut away prior to weighing samples. Tissue samples were then homogenized for 2 min. at a frequency of 30 Hz using a TissueLyser II (Qiagen) in 2 mL safe-lock tubes (Eppendorf) containing two 5 mm stainless steel beads and 1 mL of phosphate buffered saline (PBS). Homogenates were proteinase K (50 μg) treated at 50°C for one hour, centrifuged for 2 min. at 5,000g, and the supernatant transferred to a 0.45 μm filter (Millipore) tube and centrifuged for 5 min. at 10,000g. The filtered liquid was then extracted using the Qiagen All-Prep kit following manufacturer’s protocol. Isolated RNA was prepared for VirCapSeq-VERT as previously described [24]. Briefly, RNA was combined with approximately 2,000,000 oligonucleotide probes covering double stranded and single stranded DNA and RNA; (+) and (-) RNA; circular, linear and segmented viruses with a minimum sequence homology of 90% to known viruses from all 207 viral taxa known to infect vertebrates. Transcript bound probes were captured and viral RNA amplified. Sequencing was performed on the HiSeq 4000 platform (Illumina). Samples were de-multiplexed using Illumina software to generate FastQ files. The FastQ files were mapped and assembled following the protocol previously described [24]. Following identification of viral sequences through VirCapSeq-VERT, primers were designed from direct sequences and the presence of viral transcripts in diagnostic RNA libraries were confirmed by PCR. De-multiplexed high-throughput sequencing data has been deposited in the NCBI Sequencing Read Archive under the BioProject accession number PRJNA543144.

### Clinical and pathological testing

Testing for EBV in archival FFPE lymph node tissue and peripheral blood was performed at the University of Arkansas for Medical Sciences following standard clinical protocols. For archival FFPE lymph node tissue, the mean number of EBV-encoded small RNAs (EBER)-positive cells/40x high-powered fields (HPF) across 10 HPF in lymph node tissue was assessed by *in situ* hybridization and scored by a pathologist. PCR testing for EBV DNA was performed on peripheral blood from iMCD patients by ARUP Laboratories (Salt Lake City, UT, USA), where the quantitative range is 390–39,000,000 copies/mL or ViracorIBT Laboratories (Lee's Summit, MO, USA), where the assay range is 49–169,000,000 copies/mL.

## Results

**Fig 1:**
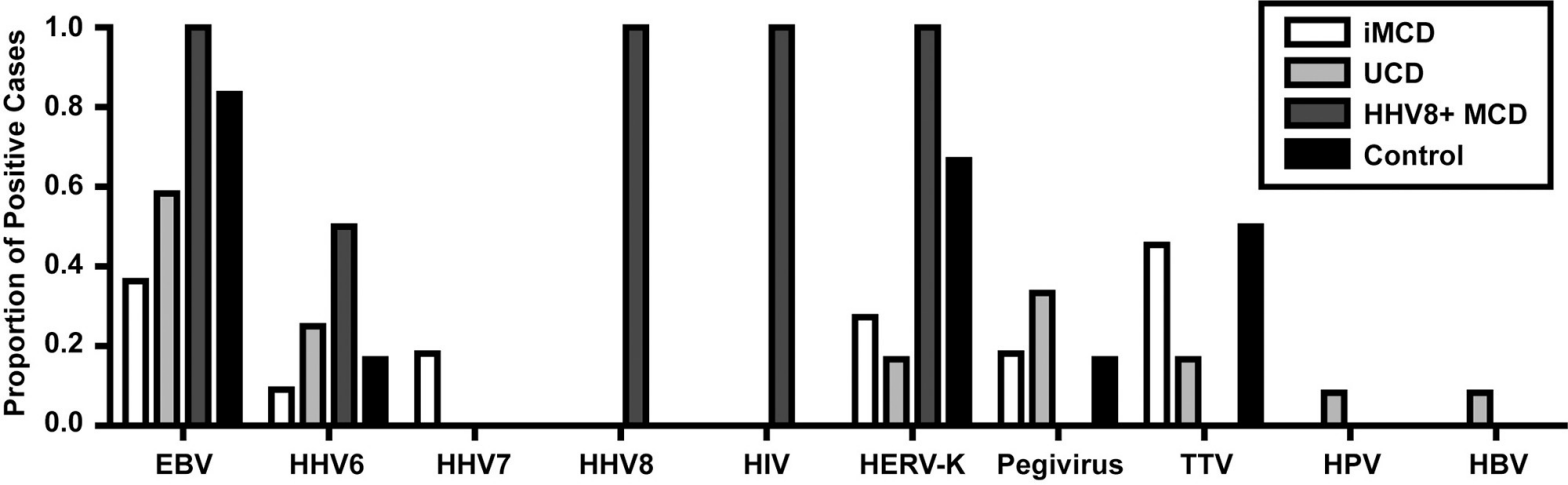
Viral transcripts detected in lymph node tissue. VirCapSeq-VERT detection of viral transcripts in fresh frozen lymph node tissue obtained from idiopathic multicentric Castleman disease (iMCD) (n = 11), unicentric CD (UCD) (n = 12), Kaposi sarcoma-associated/human herpes virus(HHV)-8 positive MCD (HHV-8+ MCD) (n = 2) and lymphoma (n = 6) patients. The proportion of cases in which transcripts for a virus were detected for each disease are presented by virus. All viruses for which transcripts were detected are presented. EBV, Epstein-Barr virus; HIV, human immunodeficiency virus; HERV-K, human endogenous retrovirus K; TTV, torque teno virus; HPV, human papillomavirus; HBV, hepatitis B virus.

To assay for known and novel viruses, as well as possible viral associations in iMCD and UCD, we performed VirCapSeq-VERT on frozen lymph node tissue from patients with these conditions. The clinical and histopathological data for all cases analyzed by VirCapSeq-VERT are presented in Table 1. As a positive control, two patients with known HIV-positive, HHV-8-positive MCD were included. Patients with Hodgkin and T cell lymphomas, in which there is no suspicion for HHV-8 infection, were included as negative controls. Consistent with results of prior routine testing, HHV-8 transcripts were not detected in any iMCD cases. The two most prevalent viruses detected in iMCD cases were torque teno virus (TTV) (5/11) and EBV (4/11) (Fig 1). Transcripts from two other members of the *Herpesviridae* family, HHV-6 and HHV-7, were detected in one and two patients respectively. In total, 5/11 iMCD cases had transcripts from *Herpesviridae* family members. Human endogenous retrovirus K (HERV-K) (3/11) and pegivirus (2/11) were also detected. No transcripts possibly-associated with a novel virus were detected in any of the iMCD cases. Of note, no viral transcripts of any sort were detected in 2/11 iMCD cases. For further reference, a full list of detected viruses for each case is listed in Table 2, including the number of viral reads, genomic coverage, and confirmation by PCR.

**Table 1:** Clinical characteristics by condition.

	iMCD	UCD	HHV-8+ MCD	Lymphoma
Number of samples	11	12	2	6
Age at diagnosis	48 ± 20	40 ± 14	45	NR
Gender	64% Male	42% Male	100% Male	NR
Relapsing disease	4/11	0/9	1/1	NA
Histopathology	6 PC, 3 HV, 2M	11 HV, 1M	2 PB	NA
CRP (mg/L)	113.2 ± 106.3 (n = 6)	0.5 ± 0.5 (n = 5)	17.5	NR
ESR (mm/hr)	81.6 ± 31.4 (n = 6)	7.4 ± 4.8 (n = 5)	57	NR
TAFRO clinical subtype	3/11	0/7	NR	NA

Clinical characteristics are presented for all samples analyzed by VirCapSeq-VERT. Histopathology specifically refers to Castleman disease histopathology subtype. PC, plasmacytic; HV, hyaline vascular; M, mixed PC and HV; PB, plasmablastic. Relapsing disease and presence of TAFRO syndrome are specific to Castleman disease. TAFRO, thrombocytopenia, anasarca, fever, reticulin myelofibrosis/renal dysfunction, organomegaly; iMCD, idiopathic multicentric Castleman disease; UCD, unicentric CD; HHV-8+ MCD, Kaposi sarcoma-associated/human herpes virus(HHV)-8 positive MCD; CRP, C-reactive protein; ESR, erythrocyte sedimentation rate. Values are presented as mean ± standard deviation, as appropriate.

**Table 2:** Summary of viral transcripts detected by VirCapSeq-VERT.

Sample	Condition	Total No.of reads	Virus(es) Identified	No. of viral reads	Genome Coverage	Confirmation (PCR)
1	Control	24561109	ND			
2	Control	14554711	Pegivirus	2	1.30%	Positive
			TTV	3	7.70%	Positive
			HERV-K	5	2.90%	Positive
			EBV	374	11.00%	Positive
3	Control	6371838	HERV-K	5	2.40%	Positive
			EBV	ND	0%	Positive
4	Control	18678639	TTV	1	2.60%	Positive
			HERV-K	19	6.60%	Positive
			EBV	2	0.10%	Positive
5	Control	24353211	TTV	1	2.60%	Positive
			EBV	ND	0%	Positive
6	UCD	9523614	TTV	1	2.60%	Positive
			EBV	1	0.10%	Positive
7	UCD	6325271	HBV	26	23.70%	Positive
			TTV	4	8.00%	Positive
8	HHV+ MCD	18307356	HIV	181	69.90%	Positive
			HERV-K	3	1.90%	Positive
			EBV	15	0.50%	Positive
			HHV-8	300	12.00%	Positive
9	HHV+ MCD	8658011	HIV	29	26.30%	Positive
			HERV-K	3	0.80%	Positive
			EBV	17	1.00%	Positive
			HHV-6	5	0.20%	Positive
			HHV-8	519	25.60%	Positive
10	iMCD	17527148	HERV-K	2	2.40%	Positive
11	UCD	22909368	Pegivirus	4	18.00%	Positive
			EBV	ND	0%	Positive
			HHV-6	ND	0%	Positive
12	UCD	22383943	EBV	4	0.20%	Positive
13	UCD	8648752	ND			
14	iMCD	15845224	TTV	47	59.30%	Positive
			EBV	1	0.10%	Positive
15	iMCD	12844820	TTV	2	5.10%	Positive
			HERV-K	5	3.20%	Positive
			EBV	1	0.10%	Positive
			HHV-7	1	0.10%	Positive
16	iMCD	14440722	TTV	1	2.60%	Positive
			HHV-6	ND	0%	Positive
			HHV-7	5	0.10%	Positive
17	Control	9173387	HERV-K	ND	0%	Positive
			EBV	1	0.10%	Positive
			HHV-6	ND	0%	Positive
18	iMCD	29439393	HERV-K	49	8.40%	Positive
19	iMCD	13550911	ND			
20	iMCD	12295074	ND			
21	iMCD	8819138	Pegivirus	1	1.10%	Positive
			EBV	7	0.10%	Positive
22	iMCD	11714699	EBV	ND	0%	Positive
23	iMCD	6553836	Pegivirus	200	46.90%	Positive
			TTV	35	14.80%	Positive
24	iMCD	9598180	TTV	2	5.10%	Positive
25	UCD	7986313	Pegivirus	30	15.70%	Positive
			HHV-6	6	0.10%	Positive
26	UCD	15354108	HPV	5	2.25%	Positive
27	UCD	12142416	EBV	ND	0%	Positive
28	UCD	9606222	HHV-6	ND	0%	Positive
29	UCD	12838304	HERV-K	32	9.10%	Positive
			EBV	ND	0%	Positive
30	UCD	7522791	Pegivirus	1	1.10%	Positive
			EBV	2	0.10%	Positive
31	UCD	15599845	Pegivirus	1	1.10%	Positive
			HERV-K	34	12.70%	Positive
			EBV	8	0.10%	Positive

For each case, clinical condition, viral transcripts, genome coverage, and results of confirmatory PCR testing are listed. For a subset of cases, some viral transcripts were not detected in the initial VirCapSeq-VERT sequencing run, but were detected in subsequent confirmatory PCR testing. For these instances, viral transcripts are listed at ND (Not Detected) with 0% genomic coverage.

Similar to iMCD, HHV-8 was not detected in any UCD cases. EBV was the most prevalent virus, detected in 7 of 12 cases (Fig 1). The next most frequent viruses identified were pegivirus (4/12) and HHV-6 (3/12), respectively. As compared to iMCD, a larger number of non-herpesviruses were detected, albeit at low prevalence: HERV-K (2/12), TTV (2/12), human papillomavirus (1/12) and HBV (1/12). No transcripts associated with a possible novel virus were detected in any of the UCD cases.

VirCapSeq-VERT detected HHV-8 and HIV transcripts in both HHV-8-positive MCD cases, confirming their known clinical statuses. EBV and HERV-K transcripts were detected in both cases as well. HHV-6 transcripts were detected in one of the two cases (Fig 1). In the Hodgkin and T cell lymphoma control cases, HHV-8 transcripts were not detected. Consistent with previous reports that both Hodgkin and T cell lymphomas associate with latent EBV infection[25–27], EBV transcripts were found in a high proportion of cases (5/6). HERV-K (4/6), TTV (3/6), HHV-6 (1/6), and pegivirus (1/6) transcripts were also detected in these lymphoma samples. These cohorts demonstrate VirCapSeq-VERT’s ability to detect transcripts from HHV-8, HIV, EBV and other known viruses.

The proportion of cases with positive findings for individual viruses across the different disease categories are shown in Fig 1. EBV was consistently detected in all groups and the most commonly detected virus, with greater than 50% of cases positive in all conditions with the exception of iMCD (36%). The other two viruses detected in all four conditions were HHV-6 and HERV-K. These viruses were found at lower prevalence than EBV in the iMCD and UCD conditions. Both pegivirus and TTV were detected in iMCD, UCD and lymphoma conditions at a prevalence of less than 50%, and were undetected in HHV-8-positive MCD. As noted above, both HIV and HHV-8 were solely detected in both HHV-8-positive MCD patients and in no other conditions.

**Fig 2:**
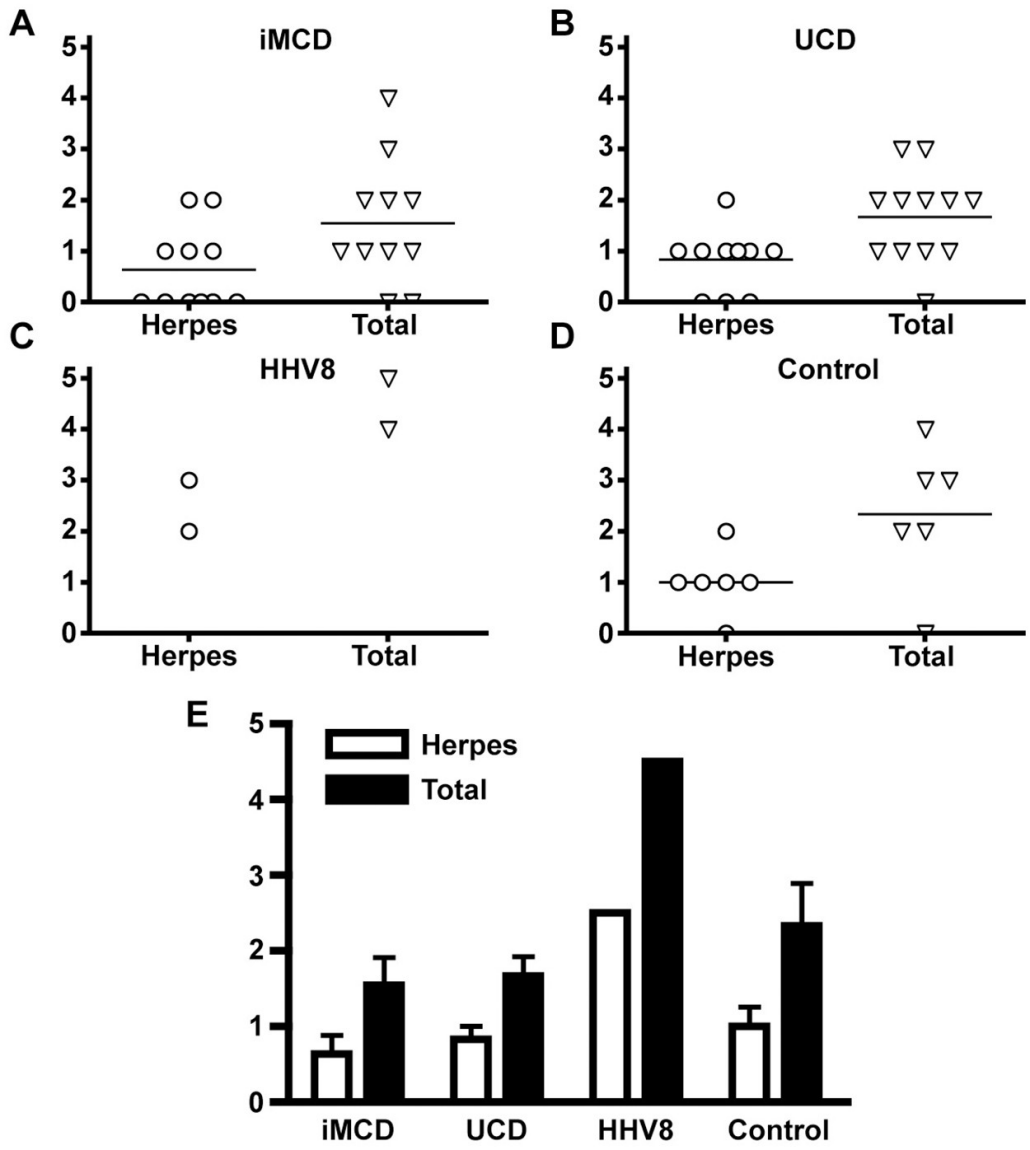
Frequency of viral transcript detection by VirCapSeq-VERT for each disease. (A-D) Number of *Herpesviridae* family members and total viruses detected by case, for each condition. (E) Average number of *Herpesviridae* family members and total viruses detected within each condition. iMCD, idiopathic multicentric Castleman disease; UCD, unicentric CD; HHV-8, Kaposi sarcoma-associated/human herpes virus(HHV)-8 positive MCD; Values are presented as mean ± standard deviation, as appropriate.

The number of different viruses detected in each sample across the different disease categories is shown in Fig 2. Overall, iMCD was associated with a lower number of different detectable viruses per patient sample, true both for members of the *Herpesviridae* family (0.64) as well as all viruses (1.55) (Fig 2A and 2E). In the UCD group, the average number of different detected herpesviruses (0.83) and total viruses (1.67) was similar to the numbers seen in iMCD (Fig 2B), with a larger prevalence of herpesvirus in large part due to increased EBV frequency. In addition to having the greatest number of *Herpesviridae* family members detected, the two HHV-8-positive MCD cases also demonstrated the greatest number of total viruses detected (Fig 2C and 2E). Lymphoma cases featured a slightly greater average number of viral associations compared to iMCD and UCD, but still reduced by a factor of ~2-fold when compared to HHV-8-positive MCD (Fig 2D and 2E).

**Fig 3:**
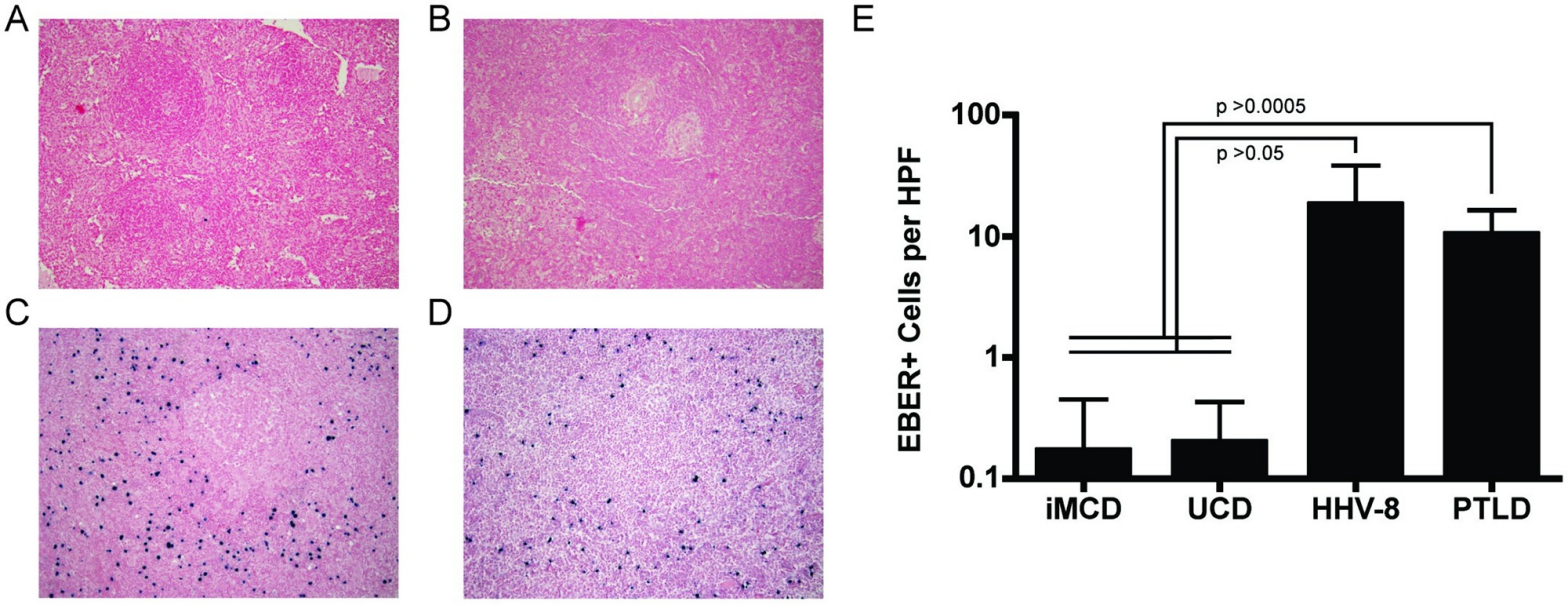
*In situ* hybridization for EBER-positive cells in lymph node tissue. (A-D) Representative images from *in situ* hybridization assays to detect Epstein-Barr virus(EBV)-encoded small RNAs (EBER) positive cells in lymph node tissue from (A) iMCD (n = 12), (B) UCD (n = 9), (C) HHV-8-positive MCD (n = 4), and (D) EBV-associated lymphoproliferative disorder (n = 4) cases. Images are shown at 10x magnification. (E) The average number of EBER positive cells per field, across ten high power fields (HPF) at 40x for each case is presented across all cases of each diagnosis. Values are presented as mean ± standard deviation, as appropriate. P-values are derived from one-sided t-test with Bonferroni correction.

Although a single virus was not universally detected across the two HHV-8-negative CD cohorts, it could be that, similar to HHV-8-positive MCD, a subset of cases within a cohort are driven by a particular virus. EBV transcripts were detected in 4/11 iMCD and 7/12 UCD cases. In addition, one case of iMCD was HHV-6 positive. Comparing iMCD cases with any detectable Herpesviridae family members (5/11) with those without such transcripts (6/11), the *Herpesviridae*-associated cases were also associated with elevated inflammatory markers, relapsing disease and TAFRO syndrome (Table 3). To investigate the correlative relationship of EBV, the most prevalent of the Herpesviridae family members, with CD subtypes including TAFRO syndrome, we assembled a separate cohort of lymph node tissue and performed *in situ* hybridization (ISH) for EBV-encoded small RNAs (EBER). We observed significantly fewer EBER-positive cells in iMCD (1.7 ± 2.8 per high power field (HPF); Fig 3A) and UCD lymph node tissue (2 ± 2.3 per HPF; Fig 3B) compared to HHV-8-positive MCD (182 ± 201.9 per HPF; Fig 3C) and EBV-associated lymphoproliferative disorder (LPD) (104 ± 60.2 per HPF; Fig 3D) lymph node tissue (*F*-statistic = 7.99; p<0.0007 for all comparisons) (Fig 3E). Additionally, clinical EBV viral load testing by PCR on peripheral blood from 29 iMCD cases (8 of which were also examined by ISH) correlated with EBER ISH results: all 29 cases, including eight iMCD-TAFRO cases, were negative.

**Table 3:** iMCD cases stratified based on detection of transcripts from the *Herpesviridae* family.

*Herpesviridae family member*:	Positive	Negative
Number of samples	5	6
CRP (mg/L)	221.5 (n = 2)	66.9 ± 70.5 (n = 4)
ESR (mm/hr)	94 (n = 2)	73.5 ± 41.3 (n = 4)
Histopathology	HV 2, M 1, PC 2	HV 1, M 1, PC 4
TAFRO	3/5	0/6
Relapsing disease	4/5	0/6
Clinical response to anti-IL-6 therapy	1/2	2/2

Clinical characteristics are presented for all iMCD patients analyzed by VirCapSeq-VERT, stratified by detection of *Herpesviridae* family member viral transcripts. Histopathology refers to Castleman disease histopathology subtype. PC, plasmacytic; HV, hyaline vascular; M, mixed PC and HV; TAFRO, thrombocytopenia, anasarca, fever, reticulin myelofibrosis/renal dysfunction, organomegaly; CRP, C-reactive protein; ESR, erythrocyte sedimentation rate. Values are presented as mean ± standard deviation, as appropriate.

## Discussion

We embarked on this study to assess for novel viral associations in CD. Given the established pathogenic role of HHV-8 in HHV-8-positive MCD, some in the field have speculated that iMCD and UCD are considered HHV-8 negative due to false negative clinical testing for HHV-8, perhaps due to epitope mutation or loss. Alternatively, it has been hypothesized that a closely related virus, known or unknown, may drive pathogenesis in iMCD and UCD. Testing these hypotheses using a high-throughput sequencing-based viral discovery platform, VirCapSeq-VERT, we were unable to identify HHV-8 in CD cases that were also negative by HHV-8 testing used routinely in the care of these patients. These findings are consistent with previous clinical observations of negative LANA-1 staining of lymph node tissue and HHV-8 PCR of peripheral blood, and provide further evidence in favor of the existence of HHV-8-negative MCD. Further, this comprehensive viral sequencing study did not identify clear associations with any previously unknown or known virus, rendering unlikely the possibility of a viral etiological driver of HHV-8-negative CD. These results fail to support the hypotheses that HHV-8 is driving disease pathogenesis despite being undetectable by clinical assays, or that another active viral infection is the etiological driver.

In the samples analyzed by VirCapSeq-VERT, we observed EBV in subsets of UCD and iMCD and were particularly intrigued by the association between *Herpesviridae* family members, notably EBV, and clinical severity of iMCD, as determined by relapsing disease and the TAFRO subtype. VirCapSeq-VERT is a highly sensitive tool for viral discovery. However, it has not been validated to determine the presence of an active EBV infection, and we found several cases for which confirmatory PCR-based testing identified EBV sequences that VirCapSeq-VERT did not (Table 2). The same observation held for HHV-6 as well. These findings indicate that PCR-based detection methods offer improved sensitivity compared to VirCapSeq-VERT, an important consideration for confirmatory testing once a potential pathogen has been identified. To determine whether associations with EBV held using routine, clinical-grade testing, we performed EBER ISH on a separate cohort of archival FFPE lymph node tissue to assess the proportion of EBER-positive cells and patterns of staining. However, no meaningful EBER staining was seen in UCD or iMCD, particularly in reference to the samples from patients with EBV-associated lymphoproliferative disorder used as a positive control for primary lymph node tissue from a clinically-associated condition driven by latent EBV reactivation. Additionally, PCR testing of peripheral blood drawn during active disease was assessed as an additional indicator of systemic infection. We did not identify clinically significant levels of EBV in yet another cohort of 29 iMCD patients that included 8 iMCD-TAFRO cases. This cohort analyzed by peripheral blood analysis had partial overlap (8/29 subjects) with the FFPE archival lymph node tissue cohort analyzed by EBER ISH. This secondary analysis was unable to confirm an association with UCD, iMCD, or the iMCD-TAFRO subtype using clinical testing methods, suggesting no latent EBV infection. Due to the rarity of iMCD and despite an international effort to collect fresh frozen or FFPE tissue together with blood samples from identical time points, the number of samples from well characterized patients in this study is sizeable compared to other similar analyses in CD[17–19]. Though larger validation cohorts, enriched for patients with iMCD-TAFRO, are needed, these analyses combine to suggest that EBV is not a pathogenic driver of CD.

VirCapSeq-VERT is a powerful and sensitive viral discovery platform capable of detecting viruses that have a minimum sequence homology of 90% to known viruses from all 207 viral taxa known to infect vertebrates. Although VirCapSeq-VERT’s breadth is wide, it could be that UCD and/or iMCD is driven by a virus that falls outside of VirCapSeq-VERT’s designated parameters (e.g., a novel virus with transcripts that share less than 90% homology with known vertebrate-infecting viruses). An increased number of unique viruses and *Herpesviridae* family members were detected in the HHV-8-positive MCD cases compared to UCD and iMCD. This finding could be explained by the immunocompromised background of HIV infection in both HHV-8-positive MCD cases. It is this background that allows HHV-8 to escape immune surveillance and initiate the cytokine storm observed in HHV-8-positive MCD. In contrast to HHV-8-positive MCD, no cases of iMCD have been reported to be HIV-positive or to occur in the context of immunosuppression. This circumstantial evidence suggests that iMCD is unlikely due to an opportunistic viral infection.

Numerous associations of unclear significance were made with non-*Herpesviridae* family viruses such as HERV-K, TTV, and pegivirus. HERV-K was detected in all conditions; however, HERV-K is incapable of generating infective virions and is widely considered to be a passenger within the human genome[28]. The frequency of detection of HERV-K sequences was greatest in the cases featuring HIV/HHV-8 co-infection or lymphoma, possibly reflecting increased transcriptional activity in a hyperproliferative state rather than a driver of disease pathology. Similar principles apply to TTV and pegivirus, both of which are viruses that have been recently-described, are widely-prevalent in human tissue, and infrequently-associated with disease[29–31]. TTV is a T-lymphotrophic virus that has received attention as a surrogate for immunocompetency, with increased TTV viremia associated with immunosuppression across many conditions. Further characterization of the human virome in health, CD, and other disease states will inform whether there may be a role for TTV or pegivirus infection in CD.

Our experimental design was based on the assumption that the viral driver of CD would be present in enlarged lymph nodes used to diagnose these cases. Given that a hallmark of CD includes characteristic histopathology in enlarged lymph nodes, the lymph node would be the central location in which one would expect to find evidence of viral infection. This principle is demonstrated by the example of HHV-8-positive MCD, where viral PCR and LANA-1 staining of lymph node tissue are considered the gold standard for evaluating HHV-8 status, more sensitive than serology or viral PCR from blood [11,12]. Additionally, HHV-8 has never been reported as found exclusively outside of the lymph node in HHV-8-postive MCD. However, it is possible that another tissue compartment, such as bone marrow, would have been more appropriate to identify viruses associated with UCD or iMCD. Additionally, our studies do not rule out the possibility that a non-viral pathogen could be driving UCD or iMCD pathogenesis. Further work is needed to examine the potential role of other types of pathogens in CD.

Since our findings suggest that viral infection is not the primary disease driver in UCD or iMCD, other previously hypothesized etiologies, such as autoimmune, autoinflammatory and paraneoplastic mechanisms may be involved. However, we are not able to rule out an indirect role for viral infection. For instance, molecular mimicry could result in a subsequent autoimmune reaction whereby an initial viral infection that initiates autoimmunity is no longer present at the time of sample analysis. Alternatively, incorporation of viral DNA into the host genome could result in gene disruption or gain-of-function of genes important for CD pathogenesis. These areas of future study will help to illuminate pathophysiology of CD and ultimately may lead to new insights that will improve treatment strategies and patient outcomes.

Our findings demonstrate that active viral infection is not the primary pathogenic driver of UCD and iMCD, in contrast to HHV8-positive MCD. Thus, distinct forms of CD exist, both with viral and non-viral etiological drivers. These observations highlight the heterogeneous nature of CD and further emphasize the importance of characterizing the unknown etiologies of UCD and iMCD to improve treatment strategies and patient outcomes.
